# COVID-19 and Diabetes: Could Diabetes Technology Research Help Pave the Way for Remote Healthcare?

**DOI:** 10.1177/1932296820929714

**Published:** 2020-05-30

**Authors:** Julia Fuchs, Roman Hovorka

**Affiliations:** 1 Wellcome Trust-MRC Institute of Metabolic Science, University of Cambridge, Cambridge, UK; 2 Department of Paediatrics, University of Cambridge, UK

**Keywords:** closed-loop, COVID-19, diabetes, diabetes technology, remote healthcare, telemedicine

*I have learned* that while a crisis poses many challenges, it can also be an opportunity to make more rapid progress than had previously seemed possible: the COVID-19 pandemic has forced us to make remote healthcare and remote research the new status quo.

As most healthcare professionals are asked to turn their attention to acute clinical care, the National Institute for Health, the Food and Drug Administration, the Medical Research Council, and the UK government have issued guidance stating that vital research should continue.^1-3^ Our research in Cambridge concerns hybrid closed-loop technology, combining real-time glucose monitoring with computer-based algorithm-directed insulin delivery to achieve glucose-responsive subcutaneous insulin delivery mimicking beta-cell function.^4^ Facilitating the more widespread adoption of new technologies, such as closed-loop insulin delivery systems, as standard of care through research is now more urgently relevant than ever. They enable greater patient independence and quality of life, reduce the need for healthcare input, simplify data sharing, and improve glycemic outcomes.^5-8^ As “routine” healthcare is turned on its head, these technologies can ease the burden on both patients and healthcare providers by facilitating remote healthcare delivery.

Significant barriers to delivering remote healthcare have been identified, including resistance to change, patient preference for face-to-face visits, concerns about patients’ health literacy, and their ability to cope with telemedicine consultations.^9^ For patients with diabetes an additional concern is the potential difficulty in uploading device data independently.

With new insulin pumps and closed-loop systems, data sharing is becoming increasingly straightforward. Many devices now allow automatic data sharing through cloud-based methods like Diasend (Glooko) and TidePool. Rather than requiring particular software or cables, patients only need to allow sharing of their data to enable remote viewing. After an initial training period, closed-loop systems require less healthcare provider input than standard therapy.^5^ Easy data viewing on handheld devices empowers patients to make independent and frequent changes to their insulin dosing as well as decreasing diabetes distress,^10,11^ and it allows healthcare providers to quickly access data when giving advice. The nature of the technology we research has enabled a relatively seamless transition to remote support during the pandemic with study participants continuing to feel well supported clinically.

What about in-person visits—in particular for training? We already used a telemedicine approach for the majority of study contacts prior to COVID-19 to minimize visit burden and have been able to build on this model to continue our research activity during the pandemic. Our study participants, with an age range of 1-80 years and from a wide range of socioeconomic backgrounds and ethnicities, have been highly flexible and motivated, readily adapting to new ways of carrying out research during the pandemic. We are conducting pump and closed-loop training by video or phone link, and participants are obtaining their own finger prick HbA1c samples using a video demonstration as guidance. Participants are readily engaging with this new methodology and report feeling empowered by their own coping skills. While some study activities have necessarily been paused, these strategies have enabled us to continue collecting vital data. Research participants are often a selective, highly motivated group of patients, but other engaged and motivated patients could manage these types of activities in combination with appropriate online training modules.

*In the future I predict* that advances in diabetes technology will be an important factor in increasing the use of telemedicine in routine diabetes care. We should welcome such change, as it saves transportation costs for patients and their families, reduces time off work and school, and integrates diabetes care into everyday life.^12^ Even in historically difficult to engage populations such as young adults, this approach has been shown to be effective.^13^ Face-to-face consultations will remain an important part of diabetes care, particularly for more complex or challenging cases. However, it is time we shift our focus to using the advances in diabetes technology to improve patient-centered care. Offering more telemedicine consultations has the potential to minimize life disruptions, increase engagement opportunities, and allows for the delivery of timely and personalized ongoing diabetes education and training.^14^ Closed-loop insulin delivery systems and other novel diabetes technologies reduce diabetes management burden for patients and healthcare providers, while also empowering patients to manage their own diabetes. These advances rely on robust research, and require the continuation of ongoing closed-loop and other diabetes technology research during the COVID-19 pandemic. With the help of this research, we can take the next step toward a more patient-centered care model, leading to lower complication rates, a decreased burden of care, and improved quality of life.
